# Supplementary material for: Identification of Freezing-Responsive microRNAs and Their Targets in Chinese Jujube by Small RNA and Degradome Sequencing
Source: Int J Mol Sci. 2026 Sep 4;27(17):7912. doi: 10.3390/ijms27177912 (PMC13566890; doi:10.3390/ijms27177912)
Supplement: Supplementary file 1 [file ijms-27-07912-s001.zip › Supplementary Material S2/novel_miR_193.pdf]

Provisional ID	:	novel_miR_193
Score total	:	1426
Score for star read(s)	:	3.9
Score for read counts	:	1418.5
Score for mfe	:	2.1
Score for randfold	:	1.6
Score for cons. seed	:	
Total read count	:	2794
Mature read count	:	2162
Loop read count	:	620
Star read count	:	12

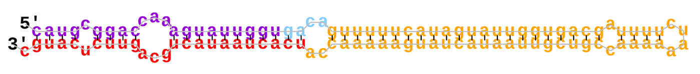

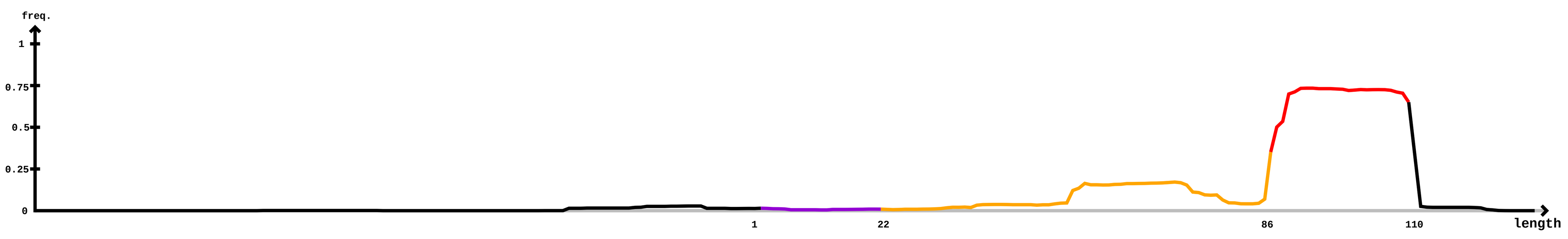

Star Mature

```
5'- gccuuuuuaacuuuggaucaauuagaggcaaauuucauggauucuaauagcagauucgguagagaaaaaaaaaauauauauauauauauauacaguccguuuauggugcggaagguccucaugcggaccaaaguauuggugacaguuuuucauaguauuggugacgauuuucuaaaaaaccgucguuaauacuaugaaaaaccaucacuaauacugcaguucucaugcggacgguucucaccacagaauu -3' obs
    gccuuuuuaacuuuggaucaauuagaggcaaauuucauggauucuaauagcagauucgguagagaaaaaaaaaauauauauauauauauauacaguccguuuauggugcggaagguccucaugcggaccaaaguauuggugacaguuuuucauaguauuggugacgauuuucuaaaaaaccgucguuaauacuaugaaaaaccaucacuaauacugcaguucucaugcggacgguucucaccacagaauu exp
```

sequence	reads	mm	sample
auggauucuaauGgcagauu	3	1	LT-2
auauauauacaguccgGuuauggu	1	1	JCK-2
auauacaguccguuuauggugcA	21	1	LCK-2
auauacaguccguuuauggugcA	22	1	LCK-1
uacaguccguuuauggugUggaag	1	1	JCK-3
uacaguccguuuauggugUggaag	2	1	JCK-2
uacaguccguuuauggugUggaag	1	1	JCK-1
guuuauggugcggaUggucc	3	1	LCK-3
guuuauggugcggaUgguccu	1	1	LT-3
guuuauggugcggaagguccuca	1	0	LT-3
guuuauggugcggaUgguccuca	5	1	LT-2
guuuauggugcggaagguccucau	1	0	LT-3
guuuauggugcggaagguccucCu	1	1	LT-3
uuuauggugcggaUgguccucaug	1	1	LT-3
uuuauggugcggaUgguccucaug	1	1	JCK-1
uuuauggugcggaUgguccucaug	1	1	JCK-3
uuauggugcggaUgguccu	2	1	LT-3
uuauggugcggaUgguccuc	4	1	JCK-2
uuauggugcggaagguccucaug	1	0	LT-3
uuauggugcggaUgguccucaugc	1	1	LT-1
uuauggugcggaagguccucaugc	2	0	LT-1
Cuauggugcggaagguccucaugc	1	1	LT-3
uuauggugcggaagguccucaugc	2	0	LT-3
uuauggugcggaUgguccucaugc	3	1	LT-3
ggugcAgaagguccucaugc	1	1	LT-2
ggugcggaagguUcucaugc	1	1	LT-1
ggugcggaagguccucaugc	1	0	LT-3
ugcggaagguccucaugc	2	0	LT-1
gcggaagguccucaugcAgacc	3	1	LT-3
ccucaugcggaccaaaUu	1	1	LT-2
cucauAcggaccaaaguauu	1	1	LT-3
ucaugcggaccaaaguauugg	1	0	JCK-2
ucaugcggacAaaaguauuggug	1	1	LT-3

	Star / Mature			
gccuuuuuaacuuuggaucaauuagaggcaaauuucauggauucuaauagcagauucgguagagaaaaaaaaaauauauauauauauauauacaguccguuuauggugcggaagguccucaugcggaccaaaguauuggugacaguuuuucauaguauuggugacgauuuucuaaaaaaccgucguuaauacuaugaaaaaccaucacuaauacugcaguucucaugcggacgguucucaccacagaauu				
....caugcggaccaaaguauuggu..	1	0	JCK-2	
....caugcggaccaGaguauuggu..	3	1	LT-2	
....caugcggaccaaaguauuggug...	1	0	LCK-2	
....caugcggaGcaaaguauuggug...	1	1	LT-2	
....caugcggacUaaaguauuggugac...	1	1	JCK-1	
....Gaugcggaccaaaguauuggugac...	1	1	LT-3	
....augcggacUaaaguauuggu..	1	1	JCK-3	
....gcggaccaaaguauuggug...	1	0	LCK-3	
....aguauuggugacaguuuuucaua..	1	0	LT-3	
....aguauuggugacaguuuuucauG..	1	1	LT-1	
....aguauuggugacaguuuuucaua..	1	0	JCK-1	
....aguauuggugacaguuuuucaua..	1	0	JCK-3	
....aguauuggugacaguuuuucauG..	2	1	LT-3	
....aguauuggugacaguuuuucaua..	1	0	JCK-2	
....aguauuggCgacaguuuuucauag...	2	1	JCK-2	
....auuggugacaguuuuucU..	1	1	LT-3	
....auuggugacaguuuuucauU..	1	1	LT-3	
....uuggugacaguuuuucaua..	1	0	LT-1	
....uggugacaguuuuucaua..	1	0	LT-3	
....ggugacaguuuuucauaguauug..	1	0	LT-1	
....ggugacaguuuuucauaguauugg...	2	0	JCK-2	
....ggugacaguuuuucauaguauugg...	1	0	JCK-3	
....caguuuuucGuaguauuggugac...	1	1	JCK-2	
....caguuuuucGuaguauuggugac...	1	1	JCK-1	
....aguuuuucGuaguauuggugac...	1	1	JCK-1	
....aguuuuucGuaguauuggugac...	3	1	JCK-2	
....aguuuuucGuaguauuggugac...	2	1	JCK-3	
....aguuuuucGuaguauuggugacg..	1	1	JCK-2	
....uuuucauaguaCuggugacgauuu..	3	1	LCK-3	
....uuucauaguauuggugacgauuuuc..	1	0	LT-1	
....uucauaguauuggugacgauu...	1	0	JCK-3	
....uucauaguauuggugacgauu...	1	0	JCK-2	
....uucauaguauuggugacgauuuucu...	1	0	LT-1	
....ucauaguaCuggugacgauuuucu...	1	1	JCK-2	
....ucauaguauuggugacgauuuucu...	2	0	LT-2	
....ucauaCuauuggugacgauuuucu...	2	1	JCK-1	
....ucauaguaCuggugacgauuuucu...	1	1	JCK-3	
....cauaguauuggugacgaAuuu...	2	1	LT-2	
....cauaguauuggugacgauuuu...	1	0	LT-2	
....cauaguaAuggugacgauuuuc..	1	1	LT-3	
....cauaguauuggugacgauuuucu...	1	0	JCK-3	
....cauaguauuggugaAgauuuucu...	4	1	LCK-3	
....cauaguauuggugacgauuuucu...	1	0	JCK-2	
....cauaguauuggugacgauuuucu...	1	0	JCK-1	
....cauaguauuggugacgauuuucAaa..	3	1	LCK-3	
....auaguauuggugacgauu...	1	0	LT-1	
....auaguaCuggugacgauuu..	1	1	JCK-3	
....auaguauuggugacgauuuu...	1	0	JCK-3	
....auaguauuggugacgauuuuc..	1	0	JCK-1	
....auaguauuggugacgauuuucu...	1	0	JCK-2	
....auaguauuggugacgauuuucu...	1	0	LT-1	
....auaguauuggugacgauuuucuG..	3	1	LT-2	
....auaguauuggugGcgauuuucuaa...	1	1	JCK-2	
....aguGuuggugacgauuuucu...	1	1	LT-1	
....aguaAuggugacgauuuucuaaa..	1	1	JCK-3	
....aguauuggugaGgauuuucuaaa..	2	1	JCK-2	
....guauuggugacgauuuucuGa...	1	1	JCK-2	
....guauuggugacgauuuucuGa...	1	1	JCK-1	
....uaAuggugacgauuuucua..	21	1	LCK-2	
....uaAuggugacgauuuucua..	17	1	LCK-1	
....uauuggugacgauuuucuaaaC...	7	1	LCK-3	
....auuggugacgauuuucuaaa..	2	0	LT-3	
....auuggugacgauuuucuaaaaa..	2	0	JCK-2	
....auuggugacgauuuucuaaaaa..	1	0	JCK-3	
....auuggugacgauuuucuaaaaaa...	3	0	LT-1	
....auuggugacgauuuucuaaaaaa...	1	0	LCK-3	
....auuggugacgauuuucuaaaaaGc...	1	1	LT-1	
....uuggugacgauuuucuaaaaG..	1	1	JCK-1	
....uuggugacgauuuucuaaaaaac..	1	0	JCK-1	
....uggugacgauuuucuaaaCa..	1	1	LT-2	

Star Mature

gccuuuuuaacuuuggaucaauuagaggcaaauuucauggauucuaauagcagauucgguagagaaaaaaaaaauauauauauauauauauacaguccguuuauggugcggaagguccucaugcggaccaaaguauuggugacaguuuuucauaguauuggugacgauuuucuaaaaaaaccgucguuaauacuaugaaaaaccaucacuaauacugcaguucucaugcggacgguucucaccacagaauu

...auuuucuGaaaaaccgucguu...	2	1	JCK-2
...auuuucuGaaaaaccgucguu...	2	1	JCK-1
...auuuucuaaaaaaaccgucguuaau...	3	0	JCK-2
...uuucuaaaaaaaccgucguuU...	1	1	JCK-2
...uuucuaaaaaaaccgucguuaau...	2	0	LT-3
...uuucuaaaaaaaccgucguuaUu...	1	1	JCK-2
...uuucuaaaaaaaccgucguuaau...	2	0	LT-1
...uuucuaaaaaaaccgucguuaUu...	1	1	LT-3
...uuucuaaaaaaaccgucguuaaua...	1	0	LT-1
...uuucuaaaaaaaccgucguuaauU...	2	1	JCK-2
...uuucuaaaaaaaccgucguuaGua...	1	1	LT-3
...uuucCaaaaaaaccgucguuaaua...	1	1	JCK-1
...uuucuaaaaaaaccgucguuaauU...	2	1	JCK-1
...uuucCaaaaaaaccgucguuaaua...	1	1	JCK-3
...uuucuaaaaaaaccgucguuaauac...	1	0	JCK-3
...uuucuaaaaaaaccgucguuaauacu...	1	0	LT-2
...uuUuaaaaaaaccgucguuaau...	2	1	LT-2
...uucuaaaaaaaccgucguuaaua...	1	0	JCK-1
...uucuaaaaaaaccgucguuaauacu...	1	0	JCK-1
...uucuaaaaaaaccgucguuaauacu...	3	0	LT-1
...uucuaaaaaaaccgucguGaauacu...	3	1	JCK-2
...uucuaaaaaaaccgucguuaauacu...	4	0	LCK-3
...uucuaaaaaaaccgucguuaauacu...	2	0	LT-2
...ucuaaaaaaaccgucguuaauacu...	4	0	JCK-3
...ucuaaaaaaaccgucguuaauacu...	1	0	JCK-1
...ucuaaaaaaaccgucguuaauacuU...	1	1	JCK-3
...ucuaaaaaaaccgucguuaauacuU...	2	1	JCK-1
...cuaaaaaaaccgucguuaG...	2	1	LT-2
...cuaaaaaaaccgucguuaa...	1	0	LT-1
...cuaaaaaaaccgucguuaa...	1	0	JCK-1
...cuaaaaaaaccgucguuaa...	1	0	LT-3
...cuaaaaaaaccgucguuaa...	7	0	LCK-3
...cuaaaaaaaccgucguuCau...	3	1	JCK-1
...cuaaaaaaaccgucguuCau...	1	1	JCK-3
...cuaaaaaaaccgucguuaGu...	8	1	LT-2
...cuaaaaaaaccgucguuaUu...	1	1	LT-3
...cuaaaaaaaccgucguuaCu...	3	1	LT-1
...cuaaaaaaaccgucguuCau...	2	1	JCK-2
...cuaaaaaaaccgucguuaGu...	2	1	LT-1
...cuaaaaaaaccgucguuaau...	12	0	LCK-3
...cuaaaaaaaccgucguuaauU...	6	1	JCK-3
...cuaaaaaaaccgucguuaauU...	5	1	JCK-2
...cuaCaaaaccgucguuaaua...	1	1	LT-2
...cuaaaaaaaccgucguuaauU...	3	1	LT-3
...cuaaaaaaaccgucguuaaua...	2	0	JCK-3
...cuaaaaaaaccgucguuaauU...	18	1	LT-2
...cuaaaaaaaccgucguuaaua...	4	0	LT-3
...cuaaaaaaaccgucguuaauU...	6	1	LT-1
...cuaaaaaaaccgucguuaauU...	31	1	LCK-3
...cuaaaaaaaccgucguuaaua...	5	0	LT-1
...cuaaaaaaaccgucguuaaua...	5	0	LT-2
...cuaaaaaaaccgucguuaaua...	17	0	LCK-3
...cuaaaaaaaccgucguuaaua...	6	0	JCK-1
...cuaaaaaaaccgucguuaauU...	2	1	JCK-1
...cuaaaaaaaccgucguuaaua...	4	0	JCK-2
...cuaaaaaaaccgucguuaauac...	2	0	LT-3
...cuaaaaaaaccgucguuaauac...	4	0	LT-2
...cuaaaaaaaccgucguuaauac...	1	0	LT-1
...cuaaaaaaaccgucguuaauacu...	16	0	LCK-3
...cuaaaaaaaccgucguuaauacu...	5	0	JCK-1
...cuaaaaaaaccgucguuaauacu...	2	0	LT-2
...cuaaaaaaaccgucguCaauacu...	1	1	LT-1
...cuaaaaaaaccgucguuaauacu...	5	0	LT-3
...cuaaaaaaaccgucguuaauacu...	1	0	LT-1
...cuaaaCaaccgucguuaauacu...	1	1	JCK-3
...cuaaaaaaaccgucguuaauacu...	7	0	JCK-2
...cuaaaaaaaccgucguuCauacu...	3	1	JCK-1
...cuaaaaaaaccgucguuaauacu...	9	0	JCK-3
...cuaaaaaaaccgucguuaGuacu...	4	1	LCK-3
...cuaaaaaaaccgucguuaGuacu...	1	1	LT-2

Star (caugcggaccaaaguauuggu) — **Mature** (ucacuaauacugcaguucucaugc)

gccuuuuuaacuuuggaucaauuagaggcaaauuucauggauucuaaauagcagauucgguagagaaaaaaaaaaauauauauauauauauauacaguccguuuauggugcggaaggucc ucaugcggaccaaaguauuggugacaguuuuucauaguauuggugacgauuuucuaaaaaaaccgucguuaauacuaugaaaaaccaucacuaauacugcaguucucaugcggacgguucucaccacagaauu

cuaaaaaaccgucguuaauacuU	3	1	LCK-3
cuaaaaaaccgucguuaauacuau	2	0	LT-3
cuaaaaaaccgucguuaauacuau	2	0	JCK-3
cuaaaaaaccgucguCaauacuau	1	1	JCK-1
cuaaaaaaccgucguuaauacuau	2	0	JCK-2
cuaaaaaaccgucguCaauacuau	1	1	JCK-3
cuaaaaaaccgucguuaauacuauU	1	1	JCK-2
uaaaaaaccgucguuaauU	4	1	LT-3
Caaaaaaccgucguuaauac	2	1	LT-2
uaaaaaaccgucguuaauacu	1	0	LT-2
uaaaaaaccgucguuaauacu	1	0	LT-3
uaaaaaaccgucguuaauacu	2	0	LT-1
uaaaaaaccgucguuaauacuUu	1	1	JCK-3
uaaaaaaccgucguuaauacuaug	5	0	JCK-3
uaaaaaaccgucguCaauacuaug	1	1	JCK-3
uNaaaaaccgucguuaauacuaug	1	1	JCK-2
Aaaaaaaccgucguuaauacuaug	5	1	JCK-1
uaaaaaaccgucguuaauacuaug	5	0	JCK-2
Aaaaaaaccgucguuaauacuaug	3	1	JCK-3
Aaaaaaaccgucguuaauacuaug	3	1	JCK-2
uaaaaaaccgucguuaauacuaug	2	0	LT-1
uaaaaaaccgucguuaauacuaug	6	0	JCK-1
uaaNaaaccgucguuaauacuaug	1	1	JCK-1
uaaaaaaccgucguCaauacuaug	1	1	JCK-1
aaaaaaccgucgCuaauacu	1	1	JCK-2
aaaaaaccgucgCuaauacu	1	1	JCK-1
aaaaaaccgucguuaauacu	2	0	JCK-1
aaaaaaccgucguuaauacua	1	0	LT-1
aaaaaaccgucguuaauacuauC	1	1	JCK-3
aaUaaaccgucguuaauacuaug	1	1	LT-1
aaUaaaccgucguuaauacuaug	1	1	JCK-2
aaaaaaccgucguuaauUcuaug	1	1	LT-3
aaaaaaccgucguuaauacuaug	9	0	JCK-2
aaaaaaccgucguuaauacuaug	6	0	JCK-1
aaaaaaccgucguuaauacuaug	2	0	LCK-3
aaaaaaccgucguuaauacuaug	6	0	JCK-3
aaaaaGccgucguuaauacuaug	2	1	JCK-1
aaaaaaccgucguuaauacuauC	3	1	JCK-2
aaaaaaccgucguuaauacuaug	8	0	LT-2
aaaaaaccgucguuaauacuaug	1	0	LT-1
aaaaaGccgucguuaauacuaug	1	1	JCK-3
aaUaaaccgucguuaauacuaug	5	1	JT-2
aaaaaaccgucguCaauacuaug	3	1	LT-1
aaaaaGccgucguuaauacuaug	1	1	JCK-2
aaaaaaccgucguuaauacuaug	1	0	LT-3
aaaaaaccgucguuaauacuauga	5	0	JCK-1
aaaaaaccgucguCaauacuauga	1	1	JCK-3
aaaaaaccgucguuaauacuauga	11	0	JCK-2
aaaaaaccgucguuaauacuauga	1	0	JT-2
aaaaaaccgucguuaauacuauga	1	0	JT-3
aaaaaaccgucguCaauacuauga	2	1	JCK-2
aaaaaaccgucguuaauacuauga	6	0	LT-1
aaUaaaccgucguuaauacuauga	1	1	LT-1
aaUaaaccgucguuaauacuauga	3	1	JCK-1
aGaaaaccgucguuaauacuauga	1	1	JCK-2
aaaaaaccgucguuaauacuauga	1	0	LT-3
aaaaaaccgucguuaauacuauga	5	0	JCK-3
aaUaaaccgucguuaauacuauga	6	1	JCK-3
aaaaaaccgucguuaauacuCuga	1	1	JCK-2
aaUaaaccgucguuaauacuauga	4	1	JCK-2
aaaaaccgucguuaauacu	1	0	JCK-3
aaaaaccgucguuaauacuau	2	0	JCK-2
aaaaaccgucguuaauacuaug	2	0	JCK-1
aaaaaccgucguuaauacuaug	2	0	JCK-2
aaaaaccgucguuaauacuaug	3	0	JCK-3
aaaaaccgucguuaauacuaug	1	0	LT-1
aaGaaccgucguuaauacuaug	2	1	LT-1
aaaaaccgucguuaauacuaugaa	1	0	JCK-3
aaaaaccgucguuaauacuaugaa	1	0	JCK-2
aaaaccgucguuaauacuauga	1	0	LT-2

Star **Mature**

```
gccuuuuuaacuuuggaucaauuagaggcaaauuucauggauucuaauagcagauucgguagagaaaaaaaaaaauauauauauauauauauacaguccguuuauggugcggaagguccucaugcggaccaaaguauuggugacaguuuuucauaguauuggugacgauuuucuaaaaaaaccgucguuaauacuaugaaaaaccaucacuaauacugcaguucucaugcggacgguucucaccacagaauu
.....aGaaccgucguuaauacuauga.....                1   1   LT-1
.....aaaaccgucguuaauacuauga.....                3   0   JCK-2
.....aaaaccgucguuaauacuauga.....                1   0   JCK-1
.....aaaccgucUuuaauacuaugaaa.....               1   1   JCK-1
.....aaccgucguuaauacuau.....                    1   0   JCK-3
.....aaccgucguuaauacuaug.....                   1   0   LT-1
.....aacGgucguuaauacuaug.....                   1   1   LT-3
.....aaccgucguuaauacuauga.....                  1   0   LT-3
.....aaccgucUuuaauacuaugaaaa.....               1   1   LT-2
.....aacGgucguuaauacuaugaaaaa.....              1   1   JCK-3
.....aaccgucguuaauacuaugaaaaa.....              1   0   LT-3
.....aacGgucguuaauacuaugaaaaa.....              1   1   JCK-2
.....accgucguuaauacuaugaaaaa.....               2   0   JCK-2
.....accgucguuaauacuaugaaaaa.....               1   0   JCK-3
.....accgucguuaauacuaugaaaaac.....              2   0   JCK-3
.....accgucguuaauacuaugaaaaac.....              4   0   JCK-2
.....accgucguuaauacuaugaaaaac.....              1   0   LT-1
.....accgucguuaauacuaugaaaaac.....              3   0   JCK-1
.....accgucguuaauacuaugaaaaaU.....              1   1   LT-3
.....Gccgucguuaauacuaugaaaaac.....              1   1   JCK-1
.....ccgucguuaauacuauga.....                    1   0   LT-3
.....ccgucguuaauacuaugaaaaacc.....              5   0   JCK-3
.....cgucguuaauacuaugaaa.....                   15  0   JT-1
.....ucguuCauacuaugaaaaaccauc.....              2   1   JCK-3
.....cguuaauacuaugaaaaaccauca.....              1   0   LT-2
.....guuaauacuaugaaaaaccauca.....               1   0   JCK-1
.....guuaauacuaugaaaaaccauca.....               2   0   JCK-2
.....guuaauacuaugaaaaaccauca.....               2   0   JCK-3
.....uuaauacuaugaaaaaccaucacu.....              1   0   LT-1
.....uaauacuaugaaaaaccaucacuC.....              1   1   LT-3
.....uaauacuaugaaaaaccaucacua.....              3   0   JCK-1
.....aauacuCugaaaaaccauc.....                   1   1   LT-3
.....Cauacuaugaaaaaccauc.....                   2   1   LT-2
.....aauacuaugaaaaaccauca.....                  1   0   LT-2
.....aauacuaugaaaaaccaucacua.....               1   0   LT-3
.....aauacuaugaaaaaccaucacua.....               1   0   JCK-3
.....aauacuaugaaaaaccaucacua.....               1   0   LT-1
.....aauacuaugaaaaaccaucacua.....               2   0   JCK-1
.....aauacuaugaaaaacUaucacua.....               1   1   LT-1
.....aauacuaugaaaaaccaucacua.....               1   0   JCK-2
.....auacuaugaaaaaccaucacuaau.....              1   0   LT-1
.....auacuaugaaaaaccaucacuaau.....              2   0   JCK-3
.....auacuCugaaaaaccaucacuaau.....              2   1   JCK-2
.....aGacuaugaaaaaccaucacuaau.....              1   1   JCK-3
.....aGacuaugaaaaaccaucacuaau.....              2   1   JCK-2
.....auacuaugaaaaaccaucacuaau.....              1   0   JCK-2
.....augaaaaaccaucacuaCuacug.....               2   1   JCK-1
.....augaaaaaccaucacuaauacugU.....              1   1   JCK-2
.....augaaaaaccaucacuaauaUugc.....              1   1   LT-1
.....augaaaaaccaucacuaauacugc.....              2   0   LT-3
.....augaaCaaccaucacuaauacugc.....              1   1   JCK-3
.....augaaaaaccaucacuaauacugc.....              2   0   LT-1
.....augaaaaaccaucacuaauacugc.....              4   0   JCK-1
.....augaaaaaccaucacuCauacugc.....              1   1   JCK-2
.....augaaaaaccaucCcuaauacugc.....              1   1   JCK-2
.....augaaaaaccaucacuaauacugc.....              3   0   JCK-3
.....Cugaaaaaccaucacuaauacugc.....              2   1   LT-2
.....augaaaaaccaucacuaauacugc.....              10  0   JCK-2
.....augaaaaaccaucacuaauacugU.....              3   1   JCK-3
.....augaaaaacUaucacuaauacugc.....              1   1   LT-2
.....augaaaaaccaucacuaauacugc.....              4   0   LCK-3
.....augaaaaacUaucacuaauacugc.....              3   1   JCK-2
.....ugaaaaaccaucacuaauacu.....                 1   0   LT-1
.....gaaaaaccaucacuaauacu.....                  1   0   LT-3
.....gaaaaaccaucacuaauacu.....                  3   0   LT-2
.....gaaaaaccaucacuaauacu.....                  1   0   LT-1
.....gaaaaaccaucacuaauacug.....                 1   0   JCK-2
.....gaaaaaccaucacuaauacug.....                 1   0   JCK-3
.....gaaaaaccaucacuaCuacug.....                 1   1   LT-1
.....gaaaaaccaucacuaCuacugc.....                2   1   JCK-1
```

Star Mature

gccuuuuuaacuuuggaucaauuagaggcaaauuucauggauucuaauagcagauucgguagagaaaaaaaaaauauauauauauauauauauacaguccguuuauggugcggaagguccucaugcggaccaaaguauuggugacaguuuuucauaguauuggugacgauuuucuaaaaaaccgucguuaauacuaugaaaaaccaucacuaauacugcaguucucaugcggacgguucucaccacagaauu

gaaaaaccaucacuaauacugc	1	0	LT-3
gaaaaaccaucacuaauacugc	2	0	JCK-1
gaaaaaccaucacuaCuacugc	1	1	JCK-2
gaaaaaccaucacuaauaUugca	1	1	LT-1
gaaaaacUaucacuaauacugcag	2	1	JCK-3
aaaaaccaucacuaauacugc	1	0	JCK-1
aaaaaccaucacuaauacugc	1	0	JCK-3
aaaaaccaucacuaauacugcagu	2	0	JCK-2
aaaaaccaucacuaauacugcagu	1	0	JCK-3
aaaaccaucacuaauacugc	2	0	LT-3
aaaaccaucacuaauacugc	1	0	LT-1
aaaaccaucacuaauacugcagu	1	0	JCK-3
aaaaccaucacuaauacugcagu	1	0	JCK-2
aaccaucacuaauacugcaguAcu	1	1	JCK-1
ccaucacuaauacugcagA	1	1	LT-3
cUaucacuaauacugcaguu	2	1	LT-2
cUaucacuaauacugcaguucuca	2	1	JCK-1
cUaucacuaauacugcaguucuca	1	1	JCK-2
caucacuaauacugcagu	1	0	JCK-2
caucacuaauacugcaguAcu	2	1	LT-3
caucacuaauacugcaguucuc	1	0	JCK-3
caucacuaauacugcaguucuc	3	0	JCK-2
caucacuaauacugcaguucuca	1	0	LT-1
caucacuaauacugcaguGcuca	1	1	LT-1
caucacuaauacuAcaguucuca	2	1	JCK-2
Uaucacuaauacugcaguucuca	1	1	JCK-1
caucacuaauaUugcaguucucau	1	1	JCK-2
cauUacuaauacugcaguucucau	3	1	JCK-1
Uaucacuaauacugcaguucucau	1	1	LT-1
Uaucacuaauacugcaguucucau	2	1	JCK-2
Uaucacuaauacugcaguucucau	1	1	JCK-3
cauUacuaauacugcaguucucau	2	1	JCK-2
aucacuaauacugcaguucucaC	3	1	JCK-3
aucacuaauacuAcaguucucau	1	1	JCK-2
auUacuaauacugcaguucucaug	1	1	JCK-2
aucacuaauacugcaguuUucaug	1	1	LT-1
auUacuaauacugcaguucucaug	1	1	JCK-3
aucacuaauacugcaguucucaug	1	0	LT-3
auUacuaauacugcaguucucaug	3	1	JCK-1
aucacuaauacugcaguucucaug	5	0	LT-1
aucacuaauacugcaguucuUaug	5	1	JCK-3
aucacuaauacugUaguucucaug	3	1	JCK-1
aucacuaauacugUaguucucaug	2	1	JCK-3
auUacuaauacugcaguucucaug	1	1	LT-3
aucacuaauacugcaguuUucaug	1	1	JCK-1
aucacuaauacugcaguucucaug	9	0	JCK-1
aucacuaauacugcaguucuGaug	3	1	LT-3
aucacuaauacugcUguucucaug	5	1	JCK-2
aucacuaauacugcaguucucaug	12	0	JCK-2
aucacuaauacugcaguucucauA	2	1	JCK-2
aucacuaauacugUaguucucaug	2	1	JCK-2
aucacuaauacugcaguucucaug	5	0	JCK-3
aucacuaauacugcaguucucauA	4	1	JCK-3
aucacuaauacugcaguucuUaug	1	1	LT-3
Uucacuaauacugcaguucucaug	2	1	LT-2
aucacuaauacugcaguucucauA	3	1	JCK-1
aucacuaauacugcaguucuUaug	2	1	JCK-2
aucacuaauacugcaguucucaugA	1	1	LT-3
aucacuaauacugcaguucucaugA	1	1	JCK-1
auUacuaauacugcaguucucaugc	1	1	LT-3
ucacuaauacugcaguuc	1	0	LT-1
ucacuaauacugcaguucuc	3	0	LT-2
ucacuaauacugcaguucuca	1	0	LT-3
ucacuaauacugcaguucucaug	1	0	JCK-2
ucacuaauacuAcaguucucaug	1	1	JCK-3
ucacuaauacugcUguucucaug	2	1	JCK-1
ucacuaauacuAcaguucucaug	1	1	JCK-2
ucacuaauacugcaguucucaug	2	0	JCK-3
ucacuaauacugcaguAcucaugc	9	1	LCK-3
ucacuaauacugcaguucucaugc	80	0	JCK-1

Sequence			
gccuuuuuaacuuuggaucaauuagaggcaaauuucauggauucuaauagcagauucgguagagaaaaaaaaaaauauauauauauauauauacaguccguuuauggugcggaagguccu **caugcggaccaaaguauuggu** (Star) gacaguuuuucauaguauuggugacgauuuucuaaaaaaccgucguuaauacuaugaaaaacca **ucacuaauacugcaguucucaugc** (Mature) ggacgguucucaccacagaauu			
…ucacuaauacugcaguucucaugc…	14	0	JT-1
…ucacuaauacugcaguAcucaugc…	19	1	LT-2
…ucacuaauacugcaguGcucaugc…	3	1	LT-1
…ucacuaauacugcaguAcucaugc…	31	1	LT-3
…ucacuaauCcugcaguucucaugc…	1	1	JT-1
…ucacuaauacugcaguGcucaugc…	2	1	JCK-2
…ucacuaauacugcaguAcucaugc…	43	1	JCK-1
…ucaUuaauacugcaguucucaugc…	1	1	LT-1
…ucacuaauacugcaguGcucaugc…	3	1	LT-3
…ucCcuaauacugcaguucucaugc…	1	1	JCK-3
…ucacuaauacugcaguucucaugc…	11	0	JT-2
…ucacuaauacugcaguucucaugc…	45	0	LT-1
…ucacuaauacuAcaguucucaugc…	1	1	LT-3
…ucacuaauacugcaguucucaugc…	37	0	LT-2
…ucacuaauacugcaCuucucaugc…	1	1	JCK-3
…ucacuaauacugcaguucucaugc…	26	0	LCK-3
…ucaUuaauacugcaguucucaugc…	3	1	LT-2
…ucacuaauacugcaguucucaugG…	1	1	LT-1
…ucacuaauacugcaguucucaugc…	47	0	LT-3
…ucacuaauacugUaguucucaugc…	6	1	JCK-2
…ucacuaauacugcCguucucaugc…	1	1	LT-2
…ucacuaauacugcaguAcucaugc…	106	1	JCK-2
…ucacuaauacugcaguAcucaugc…	64	1	JCK-3
…ucacuaauacugcaguGcucaugc…	3	1	LT-2
…ucacuaauacugcaCuucucaugc…	1	1	JCK-1
…ucacuaauacugcaguGcucaugc…	16	1	LCK-3
…ucacuaauacugcaguGcucaugc…	1	1	JCK-3
…ucacuaauacugcaguucucaugc…	7	0	JT-3
…ucacuaauacugUaguucucaugc…	4	1	JCK-3
…ucacuaauacugcaguAcucaugc…	25	1	LT-1
…ucacuaauacugcaguucucaugc…	92	0	JCK-3
…ucacuaauacugcaguAcucaugc…	9	1	JT-1
…ucacuaaCacugcaguucucaugc…	1	1	JCK-3
…ucacuaauacugcaguGcucaugc…	2	1	JCK-1
…ucacuaauacugcaguucucaugc…	135	0	JCK-2
…ucacuaauacugcCguucucaugc…	1	1	LT-3
….cacuaauacugcaguucuc…	2	0	JCK-2
….cacuaauacugcaguucuca…	4	0	LT-2
….cacuaauacugcaguucuca…	1	0	LT-3
….cacuaauacugUaguucuca…	4	1	JCK-2
….caUuaauacugcaguucuca…	1	1	LT-3
….cacuaauacugcaguucuca…	1	0	LT-1
….cacuaauacugcaguGcuca…	4	1	LT-2
….cacuaauacugUaguucucau…	1	1	LT-1
….cacuaauacugcaguucucau…	1	0	JCK-1
….cacuaauacugcaguucucau…	1	0	LT-1
….cacuaauacugUaguucucaug…	1	1	JCK-2
….cacuaauacugcaguAcucaugc…	6	1	JCK-2
….cacuaauacugcaguucucaugU…	1	1	LT-3
….cacuaauacugcCguucucaugc…	1	1	LT-3
….caUuaauacugcaguucucaugc…	7	1	LT-2
….cacuaauacugcaguucucaugc…	9	0	JT-1
….cacuaauCcugcaguucucaugc…	1	1	LT-3
….cacuaauacugcaguucucaugc…	35	0	LT-1
….cacuaauacugcaguAcucaugc…	2	1	JCK-3
….cacuCauacugcaguucucaugc…	1	1	LCK-3
….cacuaauacugUaguucucaugc…	1	1	JCK-1
….cacuaauacugcaguucuUaugc…	2	1	JCK-2
….cacuaauacugcaguucucaugc…	68	0	JCK-2
….cacuaauacugcaguucucaugc…	2	0	JT-2
….cacuaauacugcaguucucaugU…	1	1	JCK-1
….cacuaauacugcUguucucaugc…	2	1	JCK-3
….cacuaauacugcaguAcucaugc…	2	1	LT-2
….cacuaauacugcaguucucaugc…	41	0	JCK-3
….cCcuaauacugcaguucucaugc…	1	1	JCK-3
….cacuaauacugcaguucucaugc…	49	0	JCK-1
….cacuaauacugcaguAcucaugc…	2	1	LT-3
….cacuaauacugcaguGcucaugc…	3	1	LT-3
….cacuaauacugcaguAcucaugc…	2	1	LCK-3
….cacuaauacugUaguucucaugc…	4	1	JCK-2

Star **Mature**

gccuuuuuaacuuuggaucaauuagaggcaaauuucauggauucuaauagcagauucgguagagaaaaaaaaaauauauauauauauauauacaguccguuuauggugcggaagguccucaugcggaccaaaguauuggugacaguuuuucauaguauuggugacgauuuucuaaaaaaccgucguuaauacuaugaaaaaccaucacuaauacugcaguucucaugcggacgguucucaccacagaauu

cacuaauacugcaguGcucaugc	2	1	LT-1
cacuaauacugcCguucucaugc	1	1	JCK-2
cacuaauacugcaguuUucaugc	1	1	JCK-2
caUuaauacugcaguucucaugc	3	1	LT-1
caUuaauacugcaguucucaugc	3	1	LT-3
cacuaauacugNaguucucaugc	1	1	JCK-1
cacuaauacugcaguucucaugc	9	0	JT-3
cacuaauacugcaAuucucaugc	1	1	LT-2
cacuaauacugcaguAcucaugc	5	1	LT-1
cacuaauacugcaguGcucaugc	2	1	LCK-3
cacuaauacugcUguucucaugc	2	1	JCK-1
cacuaauacugcaguucuUaugc	1	1	JCK-1
cacuaauacugcaguucuUaugc	1	1	JCK-3
caUuaauacugcaguucucaugc	1	1	LCK-3
cacuaauacugcaguucucaugc	34	0	LCK-3
cacuaauacugcaguAcucaugc	1	1	JCK-1
cCcuaauacugcaguucucaugc	1	1	JCK-1
cacuaauacugcaguucucaugc	29	0	LT-3
cacuaauacugcaguucucaugc	36	0	LT-2
cacuaauacugcaguuUucaugc	2	1	JCK-3
cacuCauacugcaguucucaugc	1	1	JCK-3
cacuaauacugcaguucucCugc	1	1	LT-2
cacuaauacugcaguucucaugcg	9	0	JCK-2
cacuaauacugcaguAcucaugcg	2	1	LT-3
cacuaauacugUaguucucaugcg	9	1	JCK-3
cacuaauacugcaguucucaugcg	4	0	JCK-3
cacuaauacugUaguucucaugcg	4	1	JCK-2
cacuaauacugcaguAcucaugcg	3	1	JCK-2
cacuaauacugcaguAcucaugcg	2	1	JCK-3
cacuaauacugcaguAcucaugcg	3	1	JCK-1
cacuaauacugUaguucucaugcg	4	1	JCK-1
cacuaauacugcaguGcucaugcg	1	1	LT-1
cacuaauacugcaguucucaugUg	3	1	JCK-2
cacuaauacugcaguAcucaugcg	1	1	LT-1
cacuaauacugUaguucucaugcg	1	1	LT-1
cacuaauacugcaguucucaugcg	4	0	JCK-1
cacuaauacugcaguucucaugcg	3	0	LT-3
cacuaauacugUaguucucaugcg	1	1	LT-3
aUuaauacugcaguucuc	3	1	LT-2
acuaauacugcaguucucG	2	1	JCK-2
acuaauacugcaguucucG	2	1	JCK-1
acuaauacugcaguucucau	2	0	JCK-1
acuaauacugcaguucucau	2	0	JCK-2
acuaauacugcaAuucucaug	4	1	JCK-2
acuaauacugUaguucucaug	1	1	LT-3
aUuaauacugcaguucucaug	3	1	LCK-3
acuaauacugUaguucucaug	2	1	JCK-2
acuaauacugcUguucucaug	3	1	JCK-3
acuaauacugcaguGcucaug	1	1	JCK-2
aUuaauacugcaguucucaug	1	1	LT-1
acuaauacugcaguuUucaug	1	1	JCK-2
acuaauacugUaguucucaug	1	1	JCK-1
acuaauacugcUguucucaug	1	1	LT-1
acuaauacugcaguucucaug	2	0	LT-3
acuaauaUugcaguucucaug	2	1	JCK-1
acuaauacugcUguucucaug	2	1	JCK-2
acuaauacugcaguucucaug	2	0	JCK-2
acuaauacugUaguucucaug	8	1	JT-3
aUuaauacugcaguucucaug	1	1	LT-3
acuaauacugcaguucucaug	4	0	LT-2
acuaauacugcaguucucaug	7	0	JCK-1
acuaauacugcaguuUucaug	2	1	JCK-1
acuaauacugcaguucucaug	1	0	JCK-3
acuaauacugUaguucucaug	1	1	JCK-3
acuaauacugUaguucucaug	2	1	JT-2
aUuaauacugcaguucucaugc	2	1	LT-1
acuaauacugcaguucucaugc	4	0	LCK-3
acuaauacugcaguucucaugc	1	0	LT-3
acuaauacugcaguucucaugc	1	0	JCK-1
acuaauacugcaguGcucaugc	1	1	LT-1

```
                                                                                                                        Star                                                                                 Mature
gccuuuuuaacuuuggaucaauuagaggcaaauuucauggauucuaauagcagauucgguagagaaaaaaaaaaauauauauauauauauauacaguccguuuauggugcggaagguccucaugcggaccaaaguauuggugacaguuuuucauaguauuggugacgauuuucuaaaaaaccgucguuaauacuaugaaaaaccaucacuaauacugcaguucucaugcggacgguucucaccacagaauu
...............................................................................................................................................................................................................aUuaauacugcaguucucaugc......................   1    1    LT-2
...............................................................................................................................................................................................................acuaauacugcaguucucaugc......................   4    0    JCK-2
...............................................................................................................................................................................................................acuaauacugcaguucucaugc......................   6    0    LT-1
...............................................................................................................................................................................................................aUuaauacugcaguucucaugc......................   2    1    LT-3
...............................................................................................................................................................................................................acuaauacugcaguucucaugc......................   3    0    JCK-3
...............................................................................................................................................................................................................acuaauacugcaguucucaugc......................   5    0    LT-2
...............................................................................................................................................................................................................acuaauacugcaguGcucaugc......................   1    1    LT-3
...............................................................................................................................................................................................................acuaauacuAcaguucucaugcg.....................   1    1    JCK-3
...............................................................................................................................................................................................................acuaauacuAcaguucucaugcg.....................   1    1    JCK-2
...............................................................................................................................................................................................................acuaauacugcaguucucaugcg.....................   1    0    LT-1
...............................................................................................................................................................................................................acuaauacugUaguucucaugcgg....................   8    1    JCK-2
...............................................................................................................................................................................................................acuaauacugUaguucucaugcgg....................   1    1    JCK-1
...............................................................................................................................................................................................................acuaauacugUaguucucaugcgg....................   4    1    JCK-3
................................................................................................................................................................................................................cuaauacugcaguucuca.........................   6    0    LCK-3
................................................................................................................................................................................................................cuaauacugcaguucucaug.......................   7    0    LT-2
................................................................................................................................................................................................................cuaauacugcaguucucaug.......................   3    0    JCK-1
................................................................................................................................................................................................................cuaauacugcUguucucaug.......................   2    1    JCK-3
................................................................................................................................................................................................................cuaauacugcaguucucaug.......................   1    0    JCK-2
................................................................................................................................................................................................................cuaauacugcaguucucaug.......................   1    0    JCK-3
................................................................................................................................................................................................................cuaauacugcaguGcucaug.......................   2    1    LT-2
................................................................................................................................................................................................................cuaauacugcaguGcucaug.......................   2    1    LT-1
................................................................................................................................................................................................................cuaauacugcaguucucaug.......................   1    0    LT-3
................................................................................................................................................................................................................cuaauacugUaguucucaug.......................   3    1    JCK-2
................................................................................................................................................................................................................cuaauacugcaguucucauU.......................   2    1    LT-3
................................................................................................................................................................................................................cuaauacugcaguucucauU.......................   2    1    LT-2
................................................................................................................................................................................................................cuaauaAugcaguucucaug.......................   3    1    LT-2
................................................................................................................................................................................................................cuaauacugUaguucucaugc......................   2    1    JCK-2
................................................................................................................................................................................................................cuaauacugcaguucucaugc......................   44   0    LT-1
................................................................................................................................................................................................................cuaauacugcaguucucaugc......................   47   0    JT-1
................................................................................................................................................................................................................cuaauacugcCguucucaugc......................   1    1    JCK-2
................................................................................................................................................................................................................cuaauacugcaguucucaugc......................   50   0    LT-3
................................................................................................................................................................................................................cuaauacugcaguGcucaugc......................   7    1    LT-3
................................................................................................................................................................................................................cuaauaUugcaguucucaugc......................   1    1    JCK-3
................................................................................................................................................................................................................Uuaauacugcaguucucaugc......................   9    1    LT-2
................................................................................................................................................................................................................cuaauacugUaguucucaugc......................   2    1    JCK-3
................................................................................................................................................................................................................cuaauacugcaguucucaugc......................   34   0    JCK-3
................................................................................................................................................................................................................cuaauacugcaguucucaugc......................   60   0    JCK-2
................................................................................................................................................................................................................cuaauacugcaguGcucaugc......................   5    1    JCK-2
................................................................................................................................................................................................................cuaauacugcaguucucaugc......................   47   0    LCK-3
................................................................................................................................................................................................................Uuaauacugcaguucucaugc......................   4    1    LT-3
................................................................................................................................................................................................................Uuaauacugcaguucucaugc......................   7    1    LCK-3
................................................................................................................................................................................................................Uuaauacugcaguucucaugc......................   1    1    LT-1
................................................................................................................................................................................................................cuaauacugcaguGcucaugc......................   5    1    JCK-1
................................................................................................................................................................................................................cuaauacugcaguucucaugc......................   50   0    JCK-1
................................................................................................................................................................................................................cuaaCacugcaguucucaugc......................   1    1    LT-2
................................................................................................................................................................................................................cuaauacugUaguucucaugc......................   1    1    LT-1
................................................................................................................................................................................................................cuaGuacugcaguucucaugc......................   1    1    LT-1
................................................................................................................................................................................................................cuaauacugcaguucucaugU......................   1    1    LT-1
................................................................................................................................................................................................................cuaauacugcaguucucaugc......................   62   0    LT-2
................................................................................................................................................................................................................cuaauacugcaguGcucaugc......................   13   1    LT-2
................................................................................................................................................................................................................cuaauacugcaguGcucaugc......................   3    1    LCK-3
................................................................................................................................................................................................................cuaauacugcaguGcucaugc......................   3    1    JCK-3
................................................................................................................................................................................................................cuaauacugcaguucucaugA......................   3    1    LT-2
................................................................................................................................................................................................................cuaauacugUaguucucaugc......................   1    1    JCK-1
................................................................................................................................................................................................................cuaauacugcaguGcucaugc......................   6    1    LT-1
................................................................................................................................................................................................................cuaauacugcaguucucaugcU.....................   1    1    LT-2
................................................................................................................................................................................................................cuaauaUugcaguucucaugcgga...................   2    1    JCK-2
.................................................................................................................................................................................................................uaauacugcaguucucaugc......................   2    0    JCK-2
.................................................................................................................................................................................................................uaauacugcaguucuUaugc......................   1    1    LT-1
.................................................................................................................................................................................................................uaauacugUaguucucaugc......................   1    1    LT-1
.................................................................................................................................................................................................................uaauacugcaguucucaugc......................   4    0    LT-1
.................................................................................................................................................................................................................uaauacugcaguucucaugc......................   6    0    LT-3
.................................................................................................................................................................................................................uaauacugcUguucucaugc......................   1    1    JCK-3
.................................................................................................................................................................................................................uaauacugcaguucucaugc......................   15   0    LCK-3
.................................................................................................................................................................................................................uaauacugUaguucucaugcg.....................   2    1    JCK-2
.................................................................................................................................................................................................................uaauacugUaguucucaugcg.....................   1    1    LT-1
.................................................................................................................................................................................................................uaauacugUaguucucaugcg.....................   1    1    JCK-1
.................................................................................................................................................................................................................uaauacugcaguucucaugcg.....................   3    0    LT-1
.................................................................................................................................................................................................................uaauacugcaguGcucaugcg.....................   2    1    LT-1
..................................................................................................................................................................................................................aauacugcaguucucaug.......................   1    0    JCK-1
```

```
                                                                                     Star                                                         Mature
gccuuuuuaacuuuggaucaauuagaggcaaauuucauggauucuaauagcagauucgguagagaaaaaaaaaauauauauauauauauauacaguccguuuauggugcggaagguccucaugcggaccaaaguauuggugacaguuuuucauaguauuggugacgauuuucuaaaaaaccgucguuaauacuaugaaaaaccaucacuaauacugcaguucucaugcggacgguucucaccacagaauu
.................................................................................................................................................................................................................aauacugcaguuUucaug.......................    1    1    JCK-2
.................................................................................................................................................................................................................aauacugcaguucucaugc......................    4    0    JCK-2
.................................................................................................................................................................................................................aauacugcaguGcucaugc......................    5    1    JT-2
.................................................................................................................................................................................................................aauacugcaguucucaugc......................    4    0    LT-3
.................................................................................................................................................................................................................aauacugcaguucucaugc......................    6    0    JCK-3
.................................................................................................................................................................................................................aauacugcaguucucaugc......................    9    0    LT-1
.................................................................................................................................................................................................................aauacugcaguGcucaugc......................    4    1    LCK-3
.................................................................................................................................................................................................................aauacugcaguucucaugc......................    9    0    LT-2
.................................................................................................................................................................................................................aauacugcaguucucaugc......................    3    0    LCK-3
.................................................................................................................................................................................................................aauacugcaguucucaugc......................    3    0    JCK-1
.................................................................................................................................................................................................................aauacugcaguucucaugc......................    2    0    JT-3
.................................................................................................................................................................................................................aauacugcaguGcucaugc......................    7    1    JT-3
.................................................................................................................................................................................................................aauacugcaguGcucaugc......................    3    1    LT-3
.................................................................................................................................................................................................................aauacugcaguucucaugcg.....................    3    0    JCK-2
.................................................................................................................................................................................................................aauacugcaguucucaugcg.....................    1    0    JCK-3
.................................................................................................................................................................................................................aauacugUaguucucaugcgga...................    1    1    LT-3
..................................................................................................................................................................................................................auacugcaguucucaugc......................    3    0    LCK-3
..................................................................................................................................................................................................................auacugcaguucucaugA......................    1    1    JCK-2
..................................................................................................................................................................................................................auacugcaguucucaugc......................    3    0    JCK-3
..................................................................................................................................................................................................................auacugcaguucucaugc......................    2    0    LT-3
..................................................................................................................................................................................................................auacugcaguucucaugA......................    1    1    JCK-3
..................................................................................................................................................................................................................auacugcaguucucaugc......................    2    0    JCK-2
..................................................................................................................................................................................................................auacugUaguucucaugcgg....................    1    1    LT-1
..................................................................................................................................................................................................................auacugUaguucucaugcgga...................    1    1    JCK-2
.........................................................................................................................................................................................................................aguucucaugcggacCguucuca..........    3    1    JT-2
.........................................................................................................................................................................................................................aguucucaugcggacCguucuca..........    7    1    JT-3
.........................................................................................................................................................................................................................aguucucaugcggacCguucuca..........    15   1    JT-1
.........................................................................................................................................................................................................................aguucucaugcggacCguucucac.........    1    1    LT-3
.........................................................................................................................................................................................................................aguucucaugcggacCguucucac.........    1    1    LT-1
..........................................................................................................................................................................................................................guucucaugcggacCguucuc...........    1    1    JCK-3
..........................................................................................................................................................................................................................guucucaugcggacCguucuc...........    2    1    JCK-2
..........................................................................................................................................................................................................................guucucaugcggacCguucucac.........    5    1    LT-2
..........................................................................................................................................................................................................................guucucaugcggacCguucucacc........    1    1    LT-3
...........................................................................................................................................................................................................................uCcucaugcggacgguucu............    1    1    LT-3
...........................................................................................................................................................................................................................uCcucaugcggacgguucu............    1    1    JCK-1
...........................................................................................................................................................................................................................uCcucaugcggacgguucu............    1    1    JCK-3
...........................................................................................................................................................................................................................uucucaugcggacCguucuc...........    1    1    JCK-1
...........................................................................................................................................................................................................................uucucaugcggacCguucuc...........    1    1    JCK-3
...........................................................................................................................................................................................................................uucucaugcggacCguucuc...........    1    1    JCK-2
...........................................................................................................................................................................................................................uucucaugcggacCguucuca..........    2    1    LT-2
...........................................................................................................................................................................................................................uucucaugcggacCguucuca..........    2    1    JCK-2
...........................................................................................................................................................................................................................uucucaugcggacCguucucac.........    1    1    JCK-3
...........................................................................................................................................................................................................................uucucaugcggacCguucucacc........    1    1    LT-1
............................................................................................................................................................................................................................ucucaugcggacCguucuca..........    1    1    LT-3
............................................................................................................................................................................................................................ucucaugcggacCguucucacc........    1    1    LT-3
.............................................................................................................................................................................................................................cucaugcggacUguucucacc........    1    1    JCK-2
.............................................................................................................................................................................................................................cucaugcggacUguucucacc........    4    1    JCK-3
..............................................................................................................................................................................................................................ucaugcggacUguucucacc........    1    1    JCK-2
..............................................................................................................................................................................................................................ucaugcggacUguucucacc........    1    1    JCK-3
...............................................................................................................................................................................................................................caugcggacUguucucacc........    1    1    JCK-3
................................................................................................................................................................................................................................augcggacgguucucaccac......    1    0    JCK-2
.................................................................................................................................................................................................................................ugcggacUguucucacca.......    1    1    LT-3
.................................................................................................................................................................................................................................ugcggacUguucucacca.......    1    1    LT-1
```
